# Positive Method of Preventing and Curing Purulent Infection

**Published:** 1916-03

**Authors:** 


**Positive Method of Preventing and Curing Purulent Infection.** Chas. H. Duncan, Am. Med., Oct. 1915. This is an extremely simple method. The patient is made to lick his wounds or chew gauze saturated in the discharge if the wound cannot be conveniently reached. Specific toxins from the strain of infectious bacteria present, produce a specific protective reaction. (Note: Without intimating a contrary opinion, three suggestions occur to us. (1) It is too early to term this method positive; (2) Due credit should be given to the originators of the method—the lower animals—and we should revise the previous explanation that they survived infected wounds in spite of the addition of more or less virulent microorganisms from the saliva. (3) The regular profession should withdraw its aesthetic objections to mediaeval medicines and to the use of nosodes by homoeopaths and judge past and present questions on the basis of practical value.—Ed.).

